# CT-Based Attenuation Correction in Brain SPECT/CT Can Improve the Lesion Detectability of Voxel-Based Statistical Analyses

**DOI:** 10.1371/journal.pone.0159505

**Published:** 2016-07-21

**Authors:** Hiroki Kato, Eku Shimosegawa, Koichi Fujino, Jun Hatazawa

**Affiliations:** 1 Department of Nuclear Medicine and Tracer Kinetics, Osaka University Graduate School of Medicine, Suita, Osaka, Japan; 2 Department of Molecular Imaging of Medicine, Osaka University Graduate School of Medicine, Suita, Osaka, Japan; 3 Department of Radiology, Osaka University Hospital, Suita, Osaka, Japan; The Lee Kong Chian School of Medicine, SINGAPORE

## Abstract

**Background:**

Integrated SPECT/CT enables non-uniform attenuation correction (AC) using built-in CT instead of the conventional uniform AC. The effect of CT-based AC on voxel-based statistical analyses of brain SPECT findings has not yet been clarified. Here, we assessed differences in the detectability of regional cerebral blood flow (CBF) reduction using SPECT voxel-based statistical analyses based on the two types of AC methods.

**Subjects and Methods:**

N-isopropyl-p-[123I]iodoamphetamine (IMP) CBF SPECT images were acquired for all the subjects and were reconstructed using 3D-OSEM with two different AC methods: Chang’s method (Chang’s AC) and the CT-based AC method. A normal database was constructed for the analysis using SPECT findings obtained for 25 healthy normal volunteers. Voxel-based Z-statistics were also calculated for SPECT findings obtained for 15 patients with chronic cerebral infarctions and 10 normal subjects. We assumed that an analysis with a higher specificity would likely produce a lower mean absolute Z-score for normal brain tissue, and a more sensitive voxel-based statistical analysis would likely produce a higher absolute Z-score for in old infarct lesions, where the CBF was severely decreased.

**Results:**

The inter-subject variation in the voxel values in the normal database was lower using CT-based AC, compared with Chang’s AC, for most of the brain regions. The absolute Z-score indicating a SPECT count reduction in infarct lesions was also significantly higher in the images reconstructed using CT-based AC, compared with Chang’s AC (*P* = 0.003). The mean absolute value of the Z-score in the 10 intact brains was significantly lower in the images reconstructed using CT-based AC than in those reconstructed using Chang’s AC (*P* = 0.005).

**Conclusions:**

Non-uniform CT-based AC by integrated SPECT/CT significantly improved sensitivity and the specificity of the voxel-based statistical analyses for regional SPECT count reductions, compared with conventional uniform Chang's AC.

## Introduction

Integrated SPECT/CT provides readily available co-registered CT data for use in attenuation correction (AC) [1] and has been widely introduced in the last decade because of technical developments and innovative low-cost CT technology [2]. Compared with uniform AC, such as that obtained using Chang’s method (Chang’s-AC) based on pooled phantom data [3], the importance of non-uniform AC based on transmission scans or CT (CT-AC) during SPECT imaging of brain perfusion has been advocated in a number of previous reports [4–8]. In quantitative [4] or qualitative [5] assessments of brain perfusion SPECT, uniform AC has been reported to overestimate or underestimate the radiation absorption, leading to errors. However, the influence of non-uniform CT-AC methods on the detectability of voxel-based statistical analyses has not been clarified, although voxel-based statistical analysis of brain SPECT has become an important solution for data mining in research and clinical diagnosis. In the present study, we compared the detectability of a voxel-based Z-score analysis for lesions with decreased cerebral blood flow (CBF) between perfusion SPECT reconstructed using Chang’s-AC and that reconstructed using CT-AC. For this purpose, we made the following assumptions concerning CBF SPECT: A) an analysis with a higher specificity would likely produce a lower mean absolute Z-score for normal brain tissue, and B) a more sensitive voxel-based statistical analysis would likely produce a higher absolute Z-score for old cerebral infarction (OCI) lesions, where the CBF was severely decreased.

## Subjects and Methods

### Subjects

**Fig 1:**
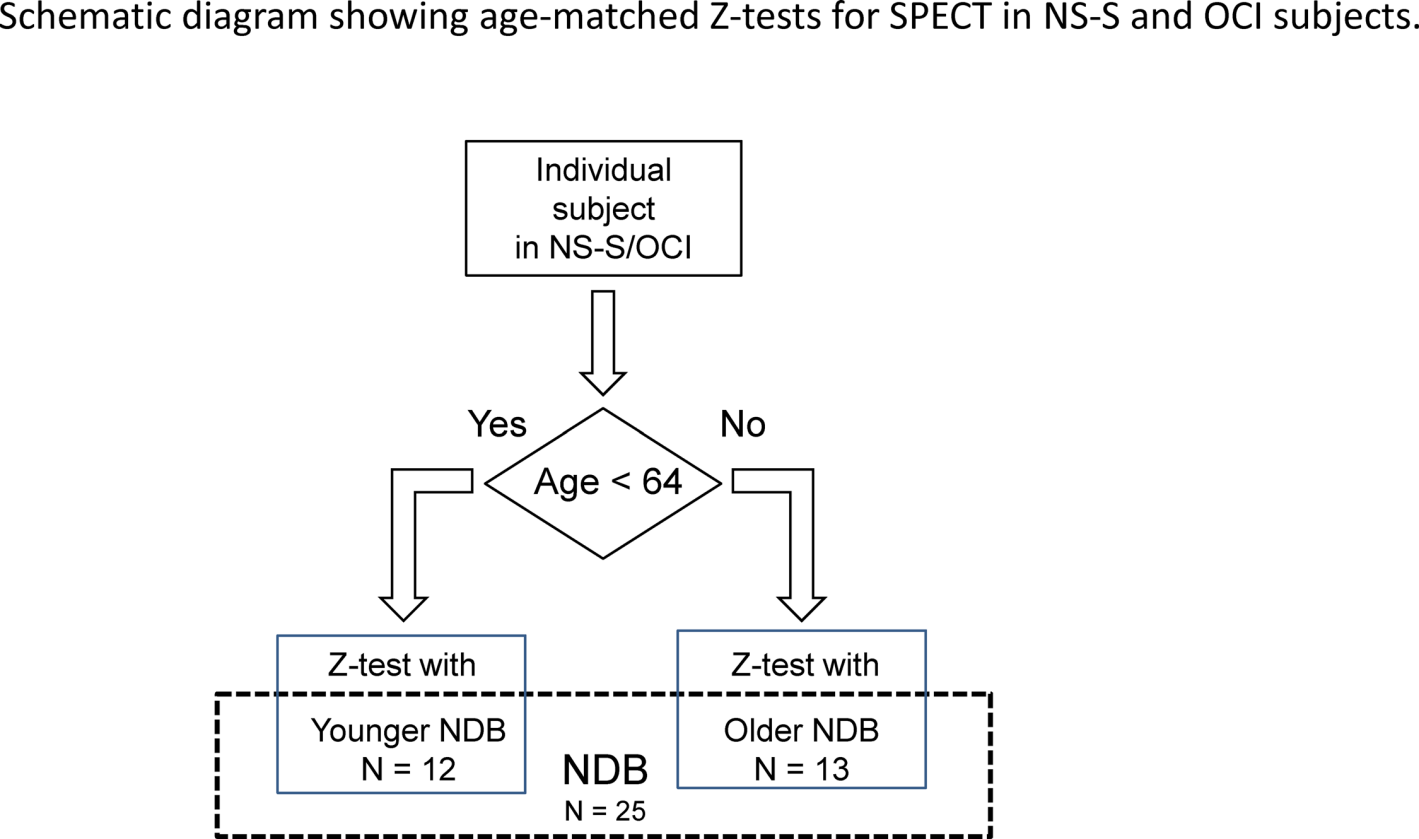
An age-matched Z-test was performed for individual NS-S subjects and/or individuals in the OCI group and the corresponding NDB subgroups; subjects younger than 64 years old were included in the younger subgroup of the NDB, while subjects who were 64 years or older were included in the older subgroup of the NDB.

Thirty-five (age, 64.7 +/- 9.0 years) healthy volunteers (HV) were recruited in the present study. All the volunteers were considered to have no history of critical medical treatment, drug abuse, alcohol indulgence, or smoking behavior. Before the present SPECT study, brain magnetic resonance imaging (MRI) and angiography (MRA) were performed using a 3.0-T whole-body superconductive scanner (Signa Excite 3.0 T; GE Yokogawa Medical Systems Ltd.). T1-weighted and T2-weighted images were obtained for all the healthy subjects. The brain MRI and MRA studies did not show any abnormal findings in any of the healthy subjects. Twenty-five subjects were randomly selected from among the HV to create a normal database (NDB). The Z-statistics between the NDB and each of the 10 remaining normal subjects (samples of normal subjects: NS-S) were calculated to evaluate the non-specific Z-score variation based on assumption A (Fig 1A).

In addition to the HV, 15 patients (age, 65.9 +/- 12.2 years) who had experienced cerebral infarctions more than three months prior to a SPECT examination were retrospectively collected. All the patients had undergone brain MRI examinations (T1-weighted, T2-weighted, fluid attenuated inversion recovery (FLAIR) images, and MRA) using a 3.0-T whole-body superconductive scanner (Signa Excite 3.0 T; GE Yokogawa Medical Systems Ltd.), as well as N-isopropyl-p-[123I]iodoamphetamine (IMP) SPECT. For all the patients, the interscan interval between the SPECT and MRI studies was less than 3 months. All the patients were stable and had not experienced any recent cerebrovascular events during the examination period.

Z-statistics between the NDB and the OCI group were calculated to assess the sensitivity of the voxel-based SPECT analysis based on the above-described assumption B (Fig 1B). In the present study, patients recruited for the OCI group had lesions where the regional CBF had been severely reduced. Considering the individual variations in radiation attenuation in the brain, we utilized the OCI group as biological brain phantom models with regional signal defects.

Each group mentioned above was divided into two subgroups according to the age at the time of SPECT acquisition to enable an age-matched Z-test (Fig 1) (Table 1).

A detailed explanation of the purpose of the study and of all the procedures used in the study was given to each of the subjects prior to their enrollment in the study. Written informed consent was obtained from each of the prospectively recruited subjects. The study was approved by the Ethical Committee of Osaka University Hospital for Clinical Research (the approval number: 15021).

**Table 1:** Subject groups and age-based subgroups.

	Younger group (<64 years)		Older group (≥64 years)		Total	
	n (M/F)	Age (SD)	n (M/F)	Age (SD)	n (M/F)	Age (SD)
NDB	12 (6/6)	57.1 (4.9)	13 (6/7)	71.8 (4.8)	25 (12/13)	64.7 (8.8)
OCI	7 (4/3)	52.5 (7.5)	8 (7/1)	74.0 (10)	15 (11/4)	65.8 (10)
Normal	5 (3/2)	56.6 (4.1)	5 (2/3)	72.4 (1.5)	10 (5/5)	64.1 (1.5)

NDB: Healthy Volunteer for Normal Database

OCI: Patients with Old Cerebral Infarction.

### SPECT acquisition

All the volunteers and the patients with OCI underwent qualitative I-123 IMP SPECT. SPECT images were acquired using an integrated SPECT/CT system (Symbia T-6; Siemens Healthcare, Erlangen, Germany). When the low-energy and high-resolution (LEHR) collimator was used, the spatial resolutions of the SPECT at a 15-mm radius were known to be 10.2 mm full-width at half maximum (FWHM) for FBP reconstruction and 4.4 mm FWHM for 3-D OSEM reconstruction, in-plane. The system resolution of planner scanning at 10 cm with the low- and medium-energy, general purpose (LMEGP) collimators and at 20% of the Tc-99m was 10.4 mm FWHM. The point-spread function (PSF) for each voxel in the reconstructed image was evaluated by modeling using a 3-D Gaussian kernel, the FWHM of which was estimated based on the distance from the original point to the interaction plane in the detector [9].

I-123 IMP (167 MBq) was first injected into the antecubital vein. Soon after the injection of the tracer, CT acquisition to obtain a CT-based mu-map was performed at 130 keV and 50 mAs. The SPECT scan was started 15 minutes after the administration of the tracer. Data was acquired for 30 min with circularly rotating gamma cameras over a 360° range in 4°-angular steps (90 views) and with 150 s/cycle and 12 repeats. The radius of rotation was 17 cm. We used a 3-D OSEM image reconstruction algorithm (Flash 3-D; Siemens Healthcare) [9–11] including transversal and axial resolution recovery and attenuation correction. For triple-energy window (TEW) scatter correction [12], 3 separate energy windows were selected for the projection data: the main window centered at 157 keV with a width of 20% (32 keV), and two additional windows below and above the main window with a width of 7% (11 keV). The iteration and subset numbers for 3-D OSEM were determined so that the balance between image convergence and statistical variance in the reconstructed images was optimized. According to our prior analysis of 3-D Hoffman phantom images, 9 iterations and 6 subsets were adopted as the reconstruction factors, providing the highest possible gray-white matter contrast and low radioactivity variation of <5% (data not shown).

In the present study, all the SPECT images were separately reconstructed using two different AC methods: Chang’s-AC, with an attenuation coefficient of 0.15 cm^-1^, and AC using built-in CT (CT-AC). These two reconstruction methods were the same except for the AC methods.

The final SPECT images were reconstructed into 3.9-mm isotropic voxels using a 128 × 128 matrix with 128 slices parallel to the orbitomeatal line. The quality of the coregistration between SPECT and CT was carefully checked to ensure the accuracy of CT-AC.

### Normal database construction

**Fig 2:**
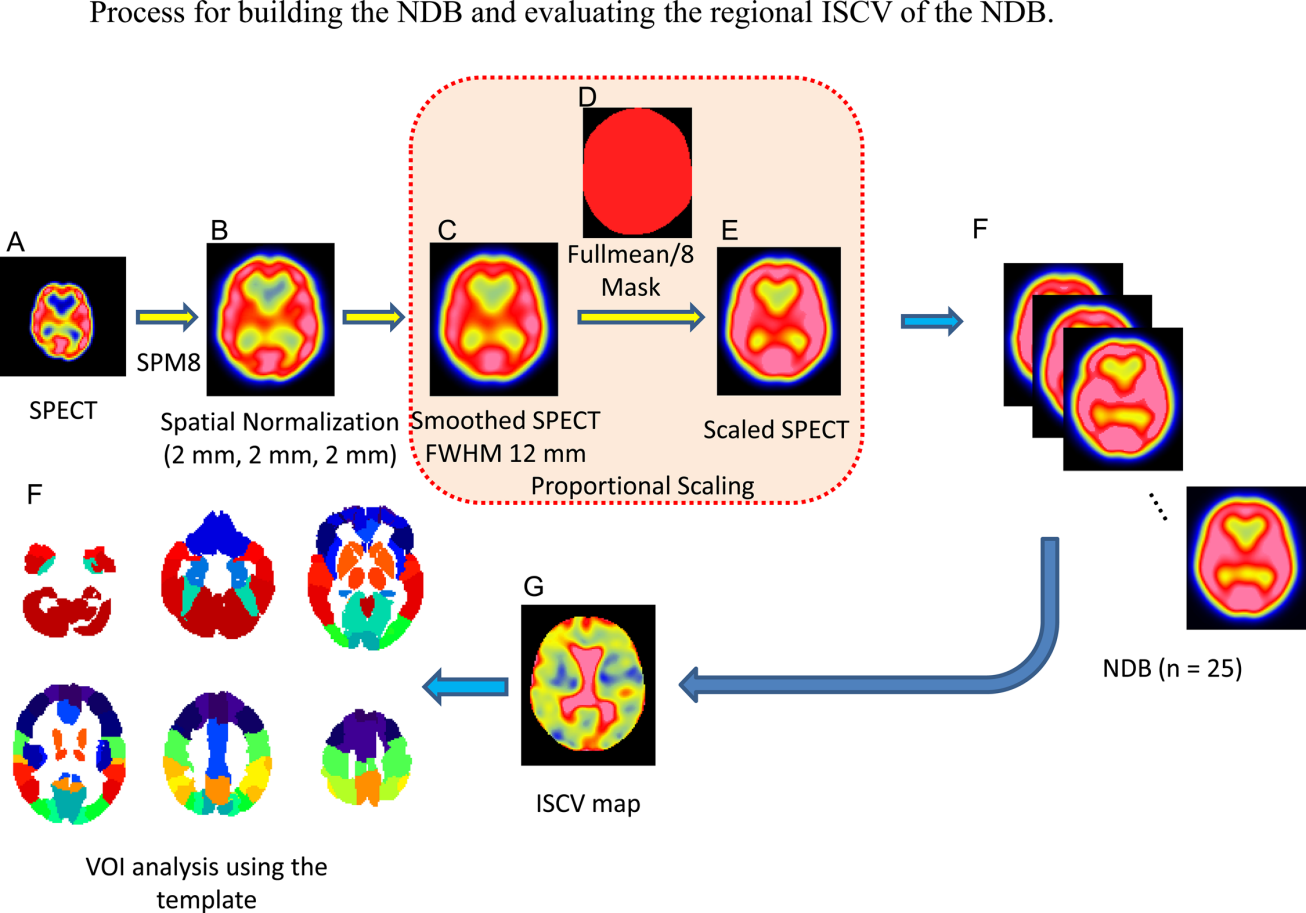
Original SPECT images (a) were anatomically normalized using SPM8. The normalized SPECT images (b) were smoothed using a Gaussian kernel with an FWHM of 12 mm. The smoothed images (c) were normalized by the global mean evaluated using the “fullmean/8 mask” (d). The scaled SPECT images (e) were used as the NDB (f). Voxel-based calculations for each NDB (Y-NDB, O-NDB) were performed to create the ISCV map. ISCV maps for the Y-NDB and the O-NDB were averaged to create the ISCV-NDB map (g). A VOI analysis of the ISCV-NDB map was performed using the template (F), based on the AAL.

Reconstructed SPECT images of HVs were spatially normalized to the Montreal Neurologic Institute (MNI) coordinates using linear coregistration and non-linear warping based on the SPECT template bundled with the Statistical Parametric Mapping 8 (SPM8) software. All the normalized SPECT images were smoothed by convolution with an isotropic Gaussian kernel of FWHM 12 mm. This filtering technique for smoothing it has been shown to be optimal [13], and FWHM of 3 times the size of voxel was practically reasonable as a rule of thumb. Proportional scaling was then performed for each image based on the global mean, which was evaluated using the “fullmean/8 mask” (Fig 2).

In this study, a total of 4 sets of NDBs were constructed: two types of NDBs were constructed based on each of the two different AC methods (NDB-Chang’s-AC and NDB-CT-AC), and each of these types of NDBs consisted of NDBs for older (O-NDB) and younger (Y-NDB) subject groups (Y-NDB-Chang’s-AC, Y-NDB-CT-AC, O-NDB-Chang’s-AC, and O-NDB-CT-AC).

### Evaluation of Z-scores

Each SPECT image was normalized, proportionally scaled, and smoothed in the same manner as for the NDBs. Absolute Z-score maps indicating the CBF decrease in each subject compared with the age-matched NDBs reconstructed using the two types of AC methods were computed using voxel-based calculations of the statistical parameters with MATLAB software Ver. 8.3.0 (The MathWorks, Inc. Natick, MA, USA).

**Fig 3:**
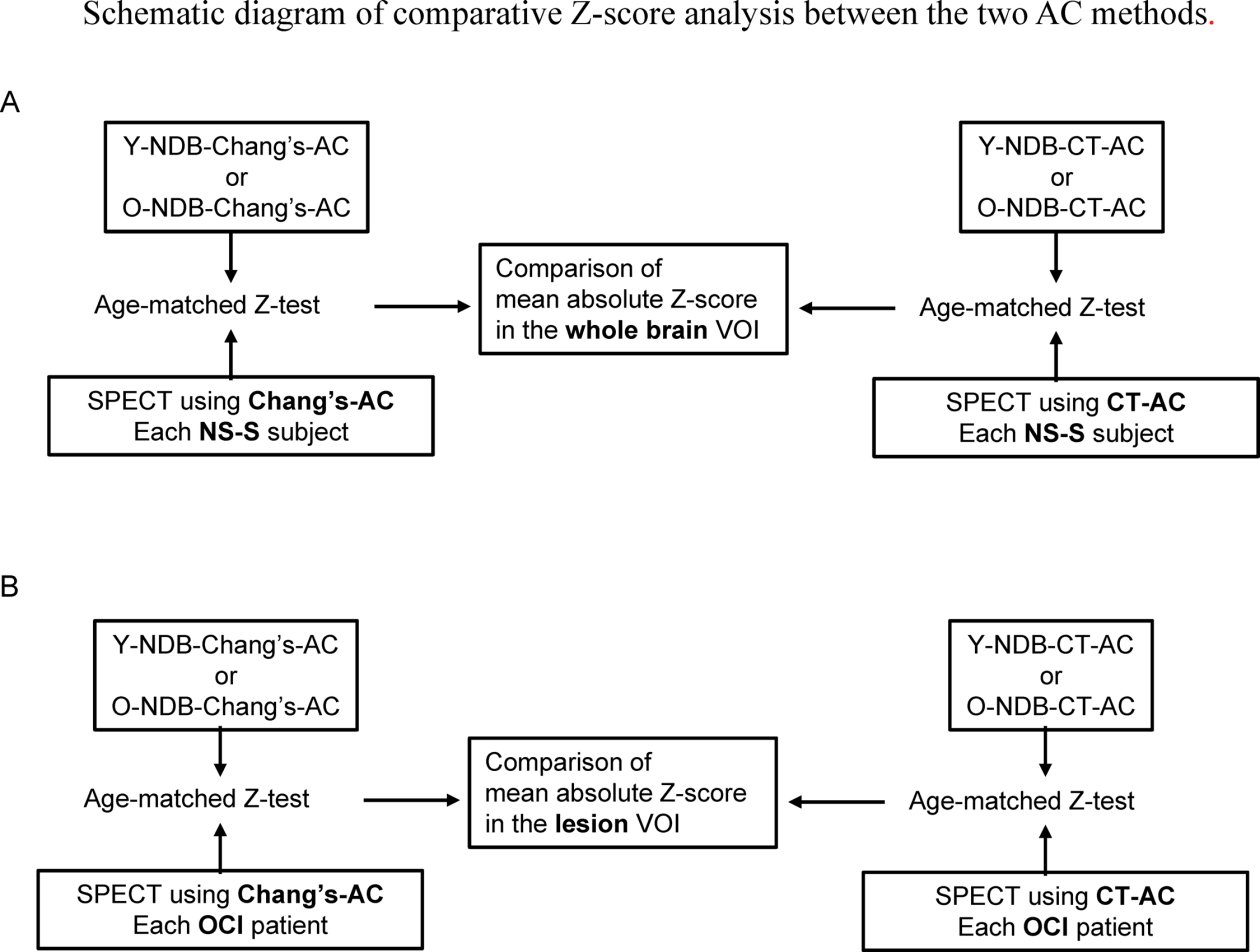
Z-statistics between each subject vs. age-matched NDB reconstructed using the same AC method was performed to yield the Z-score map. A VOI analysis for the Z-score map was performed using the whole brain VOI (A) for the NS-S or the lesion VOI for the OCI patients (B). The VOI values obtained using the two AC methods were then statistically compared. Y-NDB-Chang’s-AC: NDB reconstructed using Chang’s-AC and composed of younger subjects. O-NDB-Chang’s-AC: NDB reconstructed using Chang’s-AC and composed of older subjects. Y-NDB-CT-AC: NDB reconstructed using CT-AC and composed of younger subjects. O-NDB-CT-AC: NDB reconstructed using CT-AC and composed of older subjects.

For the Z-test between each of the NS-S and the age-matched NDB, whole brain masks with a threshold of 80% of the global mean of a group of averaged SPECT images in the NDBs were used as VOIs for Z-score comparison. The mean Z-score in the whole brain VOIs was compared using a paired nonparametric test (Wilcoxon signed-rank test) between the two AC methods in each of the NS-S (Fig 3A).

**Fig 4:**
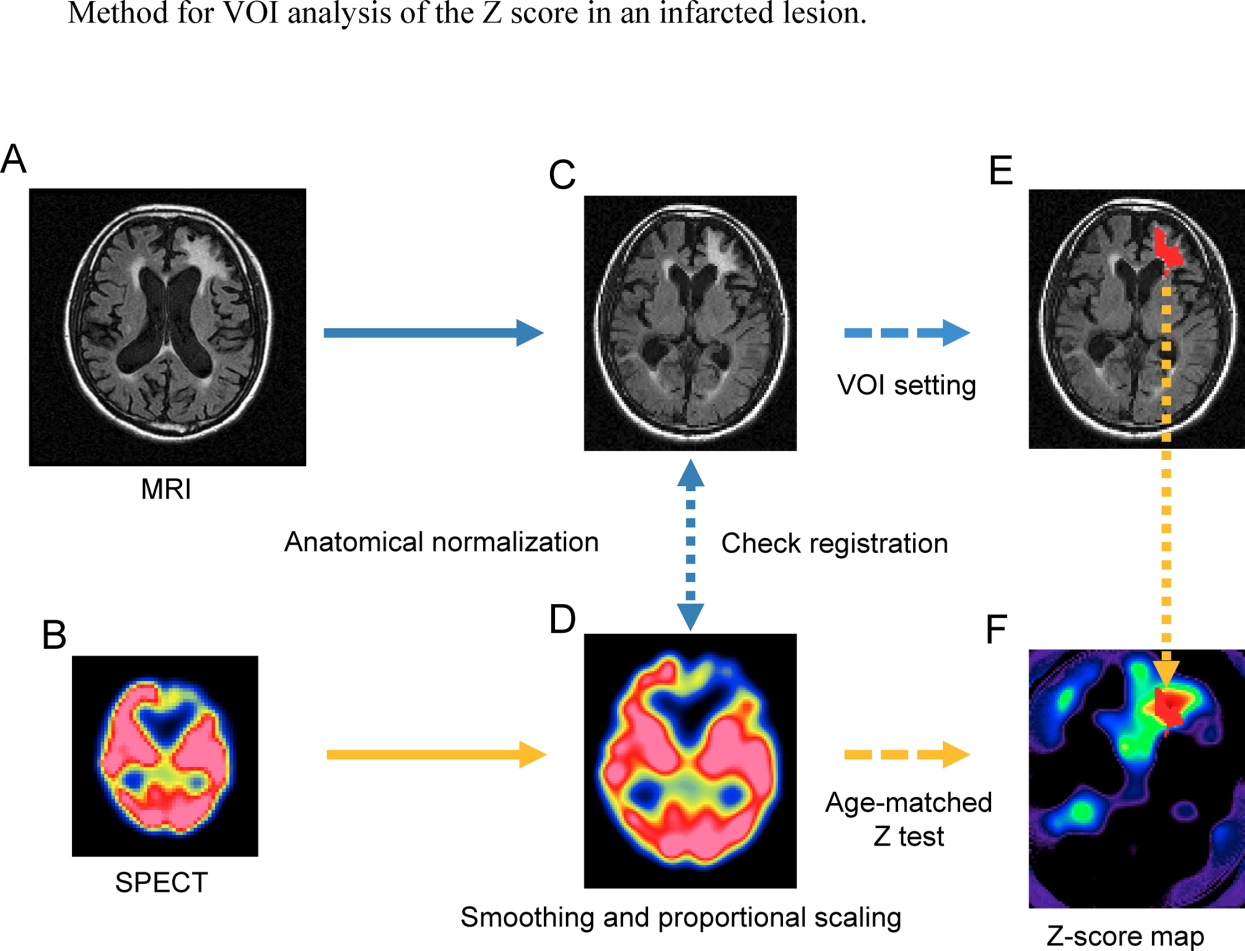
A FLAIR image (A) and a SPECT image (B) were anatomically normalized (C, D), and the accuracy of the registration between the normalized images (C) and (D) was checked. The normalized SPECT image (D) was smoothed using a Gaussian kernel (FWHM, 12 mm). The smoothed image was divided by the global mean count evaluated using the “fullmean/8 mask.” After smoothing and proportional scaling, a statistical comparison (Z test) was performed to produce the Z-score map (F). The lesion VOI (E) was created by automatically delineating the infarcted area in the normalized FLAIR image (C) based on the cut-off threshold of the FLAIR signal, which was interactively determined using software MRIcro (http://www.mccauslandcenter.sc.edu/mricro/). The lesion delineation was then visually checked and corrected independently by two experienced radiologists. The final lesion VOI was created by logical conjunction of the resultant two VOIs. The mean Z score (absolute value) in the infarcted area was measured using the lesion VOI.

In the Z-test between each OCI patient and age-matched NDB data, the T2-weighted images of the OCI patients were first spatially normalized to the MNI coordinates based on the T2 template in SPM8. The FLAIR images were also normalized using the same transfer function as that estimated in the normalization process for the T2-weighted image. The two types of SPECT images were normalized based on the SPECT template in SPM8. The precision of normalization and intermodality image registration was inspected using the “Check Registration” tool in SPM8. The volume of interest (VOI) for each infarcted lesion was made on the co-registered FLAIR image using automatic lesion delineation based on a cut-off threshold of the FLAIR signal value, which was interactively determined using a 3D ROI tool of the software MRIcro (http://www.mccauslandcenter.sc.edu/mricro/). Then, visual correction for delineation was performed independently by two experienced radiologists (Fig 4). The mean Z-score in the lesion VOI was compared using a paired nonparametric test (Wilcoxon signed-rank test) between the two AC methods in each OCI patient (Fig 3B).

### Evaluation of intersubject variation in the NDBs

The intersubject variation in each NDB was evaluated, since this parameter can influence the power of statistical inference. Voxel-based calculations of the intersubject coefficient of voxel value variation (ISCV) in the NDBs (ISCV-NDB) were performed. The resultant ISCV maps for the Y-NDB and the O-NDB were then averaged and compared between the two types of AC methods using an automated VOI analysis with an in-house tool based on a normalized VOI template, as described in a previous study [14]. The VOI template consisted of 20 VOIs that were made by combining the VOIs of the Automated Anatomic Labeling (AAL) atlas [15] (Fig 2B).

### Correlation between ISCV of radiation attenuation and ISCV-NDBs

**Fig 5:**
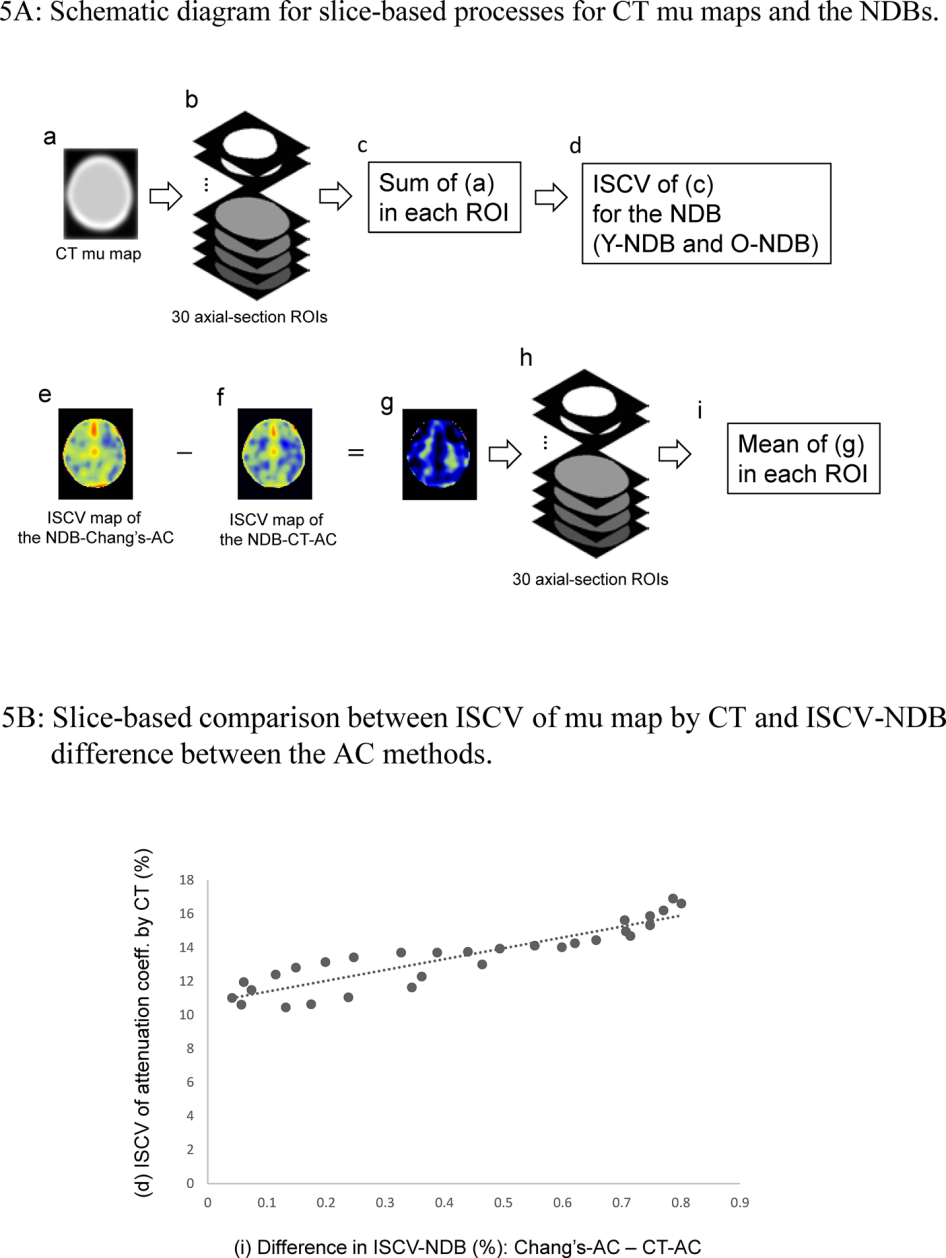
A) (a) A CT attenuation coefficient (HU) map of each subject was normalized to the transaxial template oriented along the orbitomeatal line and smoothed using an isotropic Gaussian kernel (FWHM, 12 mm). (c) The positive attenuation coefficients were summed in each slice using the brain mask ROI (b) created by masking the CT mu map (see text). (d) The ISCV of (c) for the NDB (combination of the Y-NDB and the O-NDB) was calculated. (e) The difference between the ISCV map of NDB-Chang’s-AC (e) and the ISCV map of NDB-CT-AC (f) was used to create a map of the difference in ISCV (g). The mean of (g) in each transaxial brain mask ROI (h) was then evaluated. B) The ISCV of the slice-based summation of positive attenuation coefficients (HU) using CT (d) and the mean difference in the ISCV-NDB between the two AC methods (i) for each ROI is plotted. The mean ISCV-NDB-Chang’s-AC was larger than the ISCV-NDB-CT-AC for every ROI. A significant and strong correlation was found between the two parameters.

The intersubject variation in radiation attenuation because of anatomical variation is expected to have a different impact on the ISCV-NDBs depending on the AC method that is used. To evaluate this effect, an attenuation coefficient (mu) measured using CT and the difference in ISCV-NDBs between the two AC methods were compared. The CT and SPECT images of the NDBs were spatially normalized using a transaxial template oriented along the orbitomeatal line. In the normalizing process using SPM8, the “Preserve Amount” option was specified to preserve the total amount of attenuation coefficient in the head. Brain mask ROIs were created by masking the CT image at a threshold of 0 Hounsfield Units (HU) in each transaxial plane. The rationale for choosing this threshold was that the range of head attenuation is more than 0 HU and the relationships between the measured attenuation coefficient using CT (HU) and the mu of low energy gamma rays using I-123 was defined through bilinear fitting [16]. The summation of the measured attenuation coefficient and the mean SPECT count in each ROI were evaluated in each slice from the top of the parietal lobe to the bottom of the posterior cranial fossa (Fig 5A). The difference in the ISCV of the SPECT count between the two AC methods was calculated and compared with the ISCV of the sum of the measured attenuation coefficients in each slice.

## Results

### Evaluation of Z-scores

The mean absolute value of the Z-score in the intact brain of the NS-S was significantly lower in the images that were reconstructed using the CT-AC method than in those that were reconstructed using Chang’s-AC (Wilcoxon signed-rank test, *P* = 0.005) (Table 2).

**Table 2:** Comparison of Z-score in the normal brain of NS-S between the two AC methods.

		Mean Z-score (*)	
Age	Sex	Chang's-AC	CT-AC
50	M	0.33	0.27
52	F	0.25	0.24
58	M	0.30	0.18
60	F	0.35	0.29
63	M	0.63	0.51
69	F	0.47	0.37
71	M	0.26	0.23
73	M	0.62	0.55
74	F	0.40	0.35
75	F	0.83	0.79

(*) Mean absolute value of Z-score in the whole brain VOI (brain masks thresholded at 80% of the NDB global mean).

**Fig 6:**
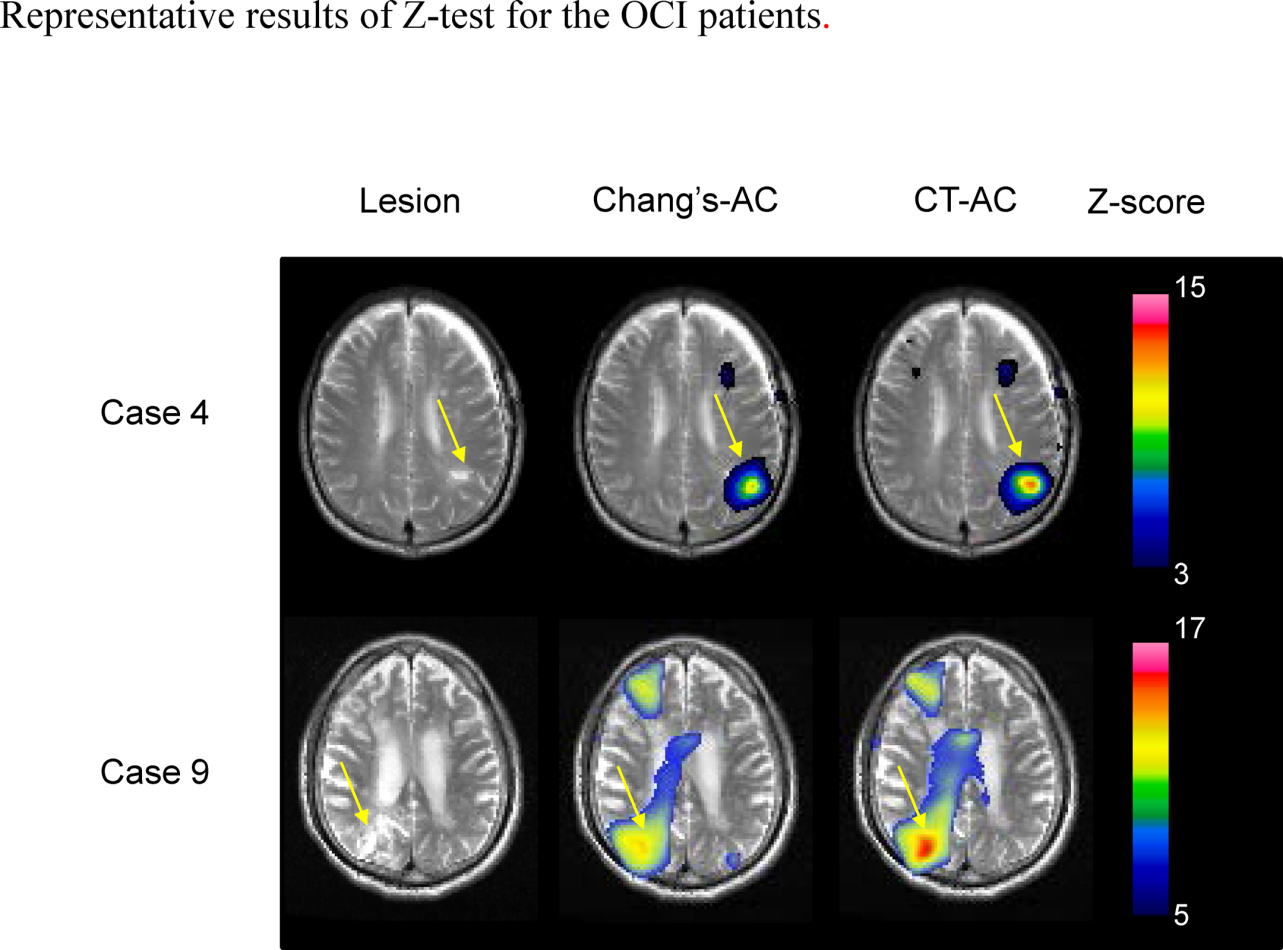
Results of Z-test for representative cases of OCI patients: (A) case 6 and (B) case 9. Lesions of each case are shown by MRI in the leftmost column. The Z-score maps by Chang’s-AC and CT-AC overlaid on the normalized MRI of each patient are displayed in the center and the rightmost column respectively.

The absolute value of the Z-score indicating the reduction in the SPECT count in infarct lesions was significantly higher in the images that were reconstructed using the CT-AC method than in those that were reconstructed using Chang’s-AC (Wilcoxon signed-rank test, *P* = 0.003) (Table 3) (Fig 6).

**Table 3:** Comparison of Z-score for OCI lesions between the two AC methods.

Case	Age	Sex	Lesion	VOI volume (CC)	Mean Z-score (*)	
					Chang’s-AC	CT-AC
1	45	F	Lt. frontal subcortical white matter	1.79	3.60	3.73
2	46	M	Rt. lateral temporal	45.17	9.69	9.77
3	54	F	Rt. lateral temporal	38.57	8.54	9.19
4	58	M	Lt. parietal, centrum semiovale	4.96	3.74	4.67
5	58	M	Lt. putamen, centrum semiovale	8.96	5.50	5.24
6	59	M	Rt. occipital	6.40	2.24	2.55
7	60	M	Rt. parietal lobule	11.18	2.85	3.11
8	64	F	Lt. centrum semiovale	1.27	1.92	2.16
9	74	M	Rt. parieto-occipital	8.54	7.70	8.43
10	74	M	Rt. parietal	4.87	5.01	5.09
11	77	M	Rt. parietal lobule	5.86	2.97	3.36
12	78	M	Lt. frontal	1.63	4.87	6.01
13	78	M	Lt. frontal	17.00	6.92	7.16
14	79	M	Rt. occipital	8.50	6.03	6.27
15	84	M	Rt. parietal subcortical white matter	1.93	2.73	3.35

(*) Mean absolute value of the Z-score for SPECT count reductions in old cerebral infarction lesions.

### Evaluation of intersubject variation in the NDBs

**Fig 7:**
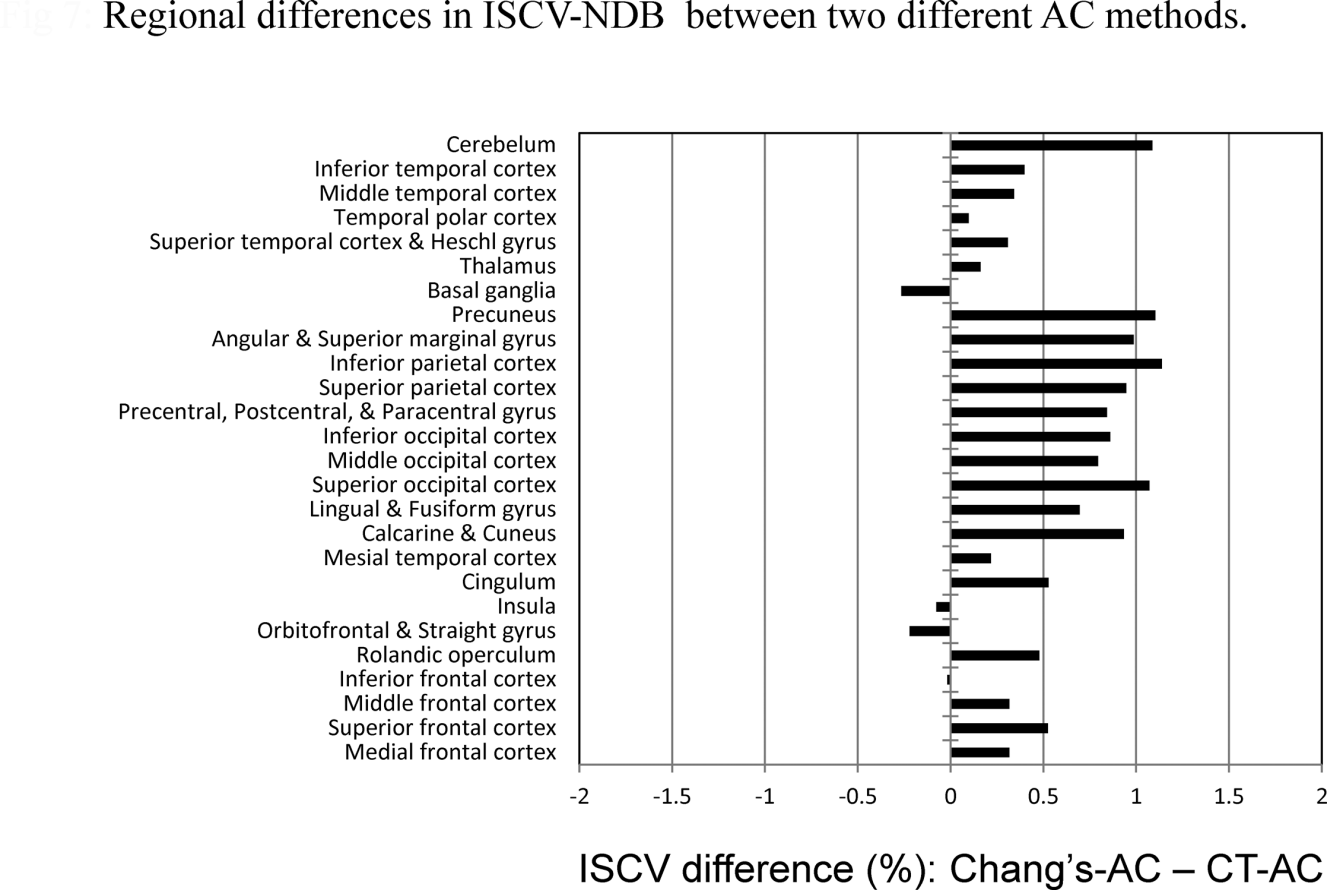
The ISCV difference (ISCV-NDB-Chang’s-AC–ISCV-NDB-CT-AC) is shown for several brain regions, each of which contains AAL elements.

The ISCV-NDB was lower using the CT-AC, compared with the conventional Chang’s method, in 90.9% of the whole brain volume except for the basal ganglia, insula, and the inferior part of the frontal cortex (9.1% of the whole brain volume) (Fig 7). This result showed that the intersubject variability of the voxel counts was higher in the NDBs reconstructed using Chang’s-AC than in those reconstructed using the CT-AC.

### Correlation between ISCV of radiation attenuation and ISCV-NDBs

As a result of the ROI analysis for CT and SPECT based on axial-section slices, the ISCV of the summation of the measured attenuation coefficient was significantly correlated with the difference in the ISCV of the mean SPECT count using Chang’s-AC, compared with CT-AC (Spearman's rank correlation coefficient: 0.934, *P* < 0.001) (Fig 5B).

## Discussion

The detection of regional abnormalities in SPECT counts using a voxel-based statistical analysis has become essential for brain SPECT. Hybrid SPECT/CT systems that enable non-uniform CT-AC and 3D-OSEM reconstruction have been gradually introduced in the field of neurological nuclear medicine. To our knowledge, this is the first study focused on the effect of non-uniform AC on sensitivity and specificity for lesion detection using a SPECT statistical analysis. By comparing the Z-scores for old ischemic lesions as calculated based on two AC methods, the absolute value of the Z-score for lesions was shown to be significantly higher when calculated based on CT-AC, compared with values calculated based on Chang’s-AC. According to our assumption, this result showed that CT-AC improved the sensitivity of Z-score analyses for CBF reduction.

The ISCV-NDB was shown to be lower in almost all the brain regions in the images obtained using CT-AC, compared with those obtained using Chang’s-AC. This difference in ISCV-NDB might have caused the difference in sensitivity in the Z-score analyses, since the Z-score varies in proportion to the reciprocal of the ISCV in the corresponding NDB. These results imply that non-pathologic variations in the SPECT count and/or noise generated through reconstruction can be suppressed using CT-AC. Actually, an age-matched Z-test between NS and the corresponding NDB showed that the mean absolute Z-score for the whole brain was lower in the images obtained using CT-AC than in those obtained using Chang’s-AC.

In an I-123 fluoropropylcarbomethoxy-3β-(4-iodophenyltropane) (FP-CIT) SPECT study [17], similar to the results of our study, the root mean squared ISCV in VOIs located in the striatal and posterior regions of a phantom and patients’ brains was lower when calculated using CT-AC, compared with the results obtained using Chang’s-AC.

In a Z-score analysis of the patients, however, the Z-score obtained using Chang’s-AC was larger than that obtained using CT-AC in a single case where the lesion was located in the putamen and centrum semiovale (Table 2, Case 5). The ISCV-NDB obtained using the CT-AC method was slightly larger than the ISCV-NDB obtained using Chang’s-AC in some regions including the central part of brain, such as the basal ganglia, insular, and inferior frontal regions (Fig 7). A previous study showed that the effect of attenuation by bone, scalp, or the head rest was smaller for the central region than for regions at the periphery of the projection, where the gamma rays must traverse the skull obliquely [7]. Thus, the Z-score in the lesion seem to be influenced by the difference in regional ISCV-NDB depending on the AC method.

The difference in the ISCV-NDB between the two AC methods was relatively remarkable in the parietal and occipital regions (Fig 7). Individual differences in the thickness of the cortical bone have been reported to be more remarkable for occipital or parietal bone than for temporal bone [18] [19]. In this study, the intersubject variability of the radiation attenuation of the head arising from anatomical variations was significantly correlated with the difference in the ISCV-NDB between CT-AC and Chang’s-AC. Thus, anatomical variation in radiation attenuation as a result of skull bone was partly compensated for using the CT-AC, possibly explaining the difference in ISCV-NDB between the two different types of AC methods.

In a study of I-123 FP-CIT SPECT, an assessment of the influence of AC on a semi-quantitative analysis for specific-to-nonspecific ratios failed to show a significant difference between the uniform and non-uniform AC methods [20, 21]. In the present study, as described above, the basal ganglia were shown to be the least affected by differences in AC-dependent variance. Dopaminergic receptor imaging, therefore, may not be strongly influenced by the choice of AC method.

The present study has some limitations. First, our analysis involved only one apparatus and only one reconstruction method to clarify the differences in NDBs and the detectability of lesions between the two different AC methods. Although OSEM reconstruction in our SPECT scanner has been shown to be more accurate than filtered back projection method for quantitative CBF measurements [4], a multicenter study involving many other SPECT scanners is needed to establish firm evidence. Second, because the demarcation of the head contour in Chang’s-AC method and/or registration checks for the SPECT and CT images depend on the SPECT operator, this process could have caused a certain amount of noise in the NDBs [17]. Although experienced radiological technologists performed the reconstructions in this study, this type of noise is practically inevitable. Thirdly, in the anatomical normalization process of the OCI brain SPECT images, the remarkable signal reductions in the lesions could have caused a slight misregistration of the SPECT findings with the MNI template, although the lesion volumes were relatively small and this effect seemed to be limited.

## Conclusions

The sensitivity of the detection of regional SPECT count reductions using a voxel-based statistical analysis was shown to be significantly improved using CT-AC by integrated SPECT/CT, compared with the conventional Chang’s-AC method. Although the improvement in the Z-score was relatively small, the CT-AC method can suppress non-specific variations in NDBs—especially for the parietal and occipital regions—possibly influencing the diagnosis of cognitive impairments including Alzheimer disease or dementia with Lewy bodies.
